# Preoperative squamous cell carcinoma antigen and albumin serum levels predict the survival of patients with stage T1-3N0M0 esophageal squamous cell carcinoma: a retrospective observational study

**DOI:** 10.1186/s13019-020-01163-6

**Published:** 2020-05-26

**Authors:** Lei-Lei Wu, Xuan Liu, Wei Huang, Peng Lin, Hao Long, Lan-Jun Zhang, Guo-Wei Ma

**Affiliations:** Department of Thoracic Surgery, Sun Yat-sen University Cancer Center, State Key Laboratory of Oncology in South China, Collaborative Innovation Center for Cancer Medicine, 651 Dongfeng East Road, Guangzhou, 510060 P. R. China

**Keywords:** Squamous cell carcinoma antigen, Albumin, Cancer-specific survival, Esophageal squamous cell cancer

## Abstract

**Background:**

This study aimed to explore the significance of preoperative levels of squamous cell carcinoma antigen (SCC-Ag) and albumin on the cancer-specific survival (CSS) of patients with stage T1-3N0M0 in esophageal squamous cell cancer (ESCC).

**Methods:**

The data of 308 patients who underwent esophagectomy between 1996 and 2011 were analyzed. SCC-Ag and albumin levels were measure 1 week before surgery. The optimal cutoff levels of SCC-Ag and albumin were determined using the X-Tile software, which were 1.0 μg/L and 39.8 g/L, respectively. The associations between SCC-Ag and albumin levels and clinicopathological characteristics were assessed using the χ^2^ test, Student’s t-test and Fisher’s exact test. Cox univariable and multivariable analyses were computed to identify SCC-Ag and albumin levels as independent prognostic factors related to the CSS of patients with ESCC. We used the Kaplan-Meier survival curve to determine the significance of SCC-Ag and albumin level on ESCC in the long-term follow-up.

**Results:**

The 5-year CSS rate for the entire cohort was 65.0%. There was a significant difference in CSS between the low and high SCC-Ag level groups (hazard ratio [HR], 1.828, 95% confidence interval [CI], 1.203–2.778; *P* = 0.005). Patients with ESCC with low albumin level had a worse CSS than those with high albumin level (HR, 0.540; 95% CI, 0.348–0.838; *P* = 0.006). Patients with both high SCC-Ag and low albumin levels had worse 5-year CSS than patients with low SCC-Ag and high albumin levels (*P* < 0.05).

**Conclusions:**

Preoperative serum SCC-Ag and albumin levels can predict survival in patients ESCC with stage T1-3N0M0. Patients with ESCC with high SCC-Ag and low albumin levels may have a poor survival outcome.

## Background

Esophageal cancer ranks seventh among the most common cancers and sixth among malignancy-related mortality in men worldwide, especially in East Asia, of which more than half of the total cases globally are of Chinese origin [1, 2]. Contrary to Western countries, in China, esophageal squamous cell cancer (ESCC) accounts for > 90% of esophageal cancer cases [3, 4]. Despite the application of advanced surgical techniques, radiotherapy, and chemotherapy, the recurrence of ESCC remains inevitable and leads to poor prognosis [5].

Previous studies suggested that serum squamous cell carcinoma antigen (SCC-Ag) levels had likely an effect on prognosis of malignant tumor [6–9], such as oral squamous cell carcinoma [7, 10] and head and neck cancer [11, 12]. Indeed, SCC-Ag is a widely used tumor marker in oncology. In addition, the findings of some studies revealed that serum albumin level might be related to the survival outcomes of patients with cancer, such as ovarian cancer [13] and endometrial cancer [14]. However, the significance of preoperative serum albumin levels is still unclear in the prognosis of patients with ESCC.

With regard to ESCC, existing literatures identified high SCC-Ag level as a predictor of poor prognosis [6, 15]. Shimada et al. collected the medical records of 309 patients who underwent esophagectomy and analyzed the data of these patients with ESCC. They found that patients with high SCC-Ag level had worse survival (*P* < 0.05) [15]. In addition, Cao also validated the results of Shimada’s study by analyzing the data of 379 patients with ESCC [6]. The two abovementioned studies included patients with metastasis of the lymph nodes (LNs).

This study aimed to investigate the exact value of SCC-Ag and albumin levels on the prognosis of patients with ESCC in stage T1-3N0M0. Patients with metastasis of the LNs were excluded because these patients would have better survival by receiving neoadjuvant therapy based on the results of Hong Yang’s clinical trial [16].

## Methods

### Patients and follow-up

**Fig. 1:**
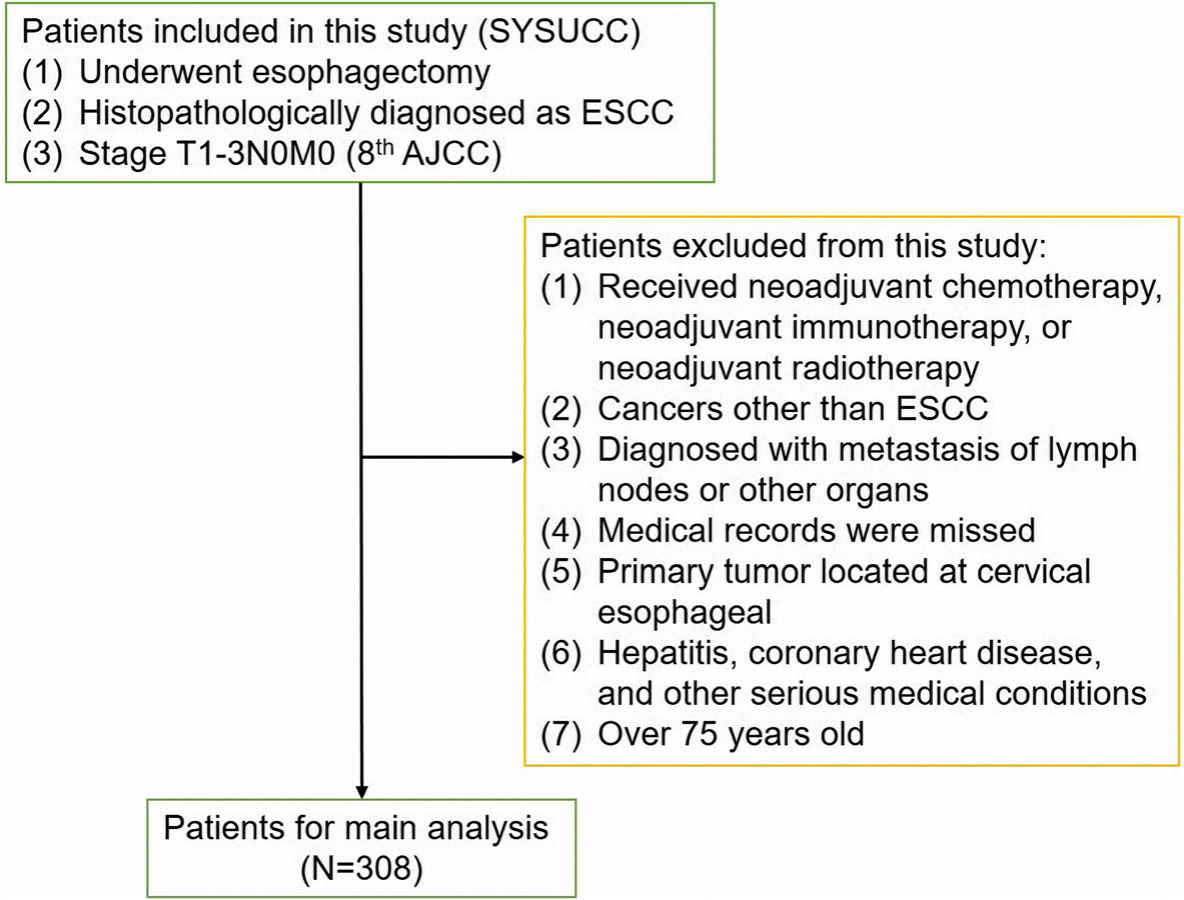
Flowchart of the study

This retrospective study was approved by the ethics committee of Sun Yat-sen University Cancer Center (SYSUCC; Approval No. YB2016–070). Eligible cases, screened from the database of the Department of Thoracic Surgery in the SYSUCC, were defined as patients with ESCC histopathologically diagnosed as stage T1-3N0M0 after esophagectomy between 1996 and 2011. Patients with the following conditions were excluded: (1) neoadjuvant chemotherapy, neoadjuvant immunotherapy, or neoadjuvant radiotherapy; (2) cancers other than ESCC; (3) diagnosis of metastasis of LNs or other organs; (4) age > 75 years; (5) missing medical records; (6) primary tumor located at cervical esophagus; and (7) hepatitis, coronary heart disease, and other serious medical conditions. All patients were restaged based on the American Joint Committee on Cancer Staging Manual, eighth edition [17, 18]. Finally, a total of 308 patients were enrolled in our study (Fig. 1).

We recommend that patients visit the outpatient department for follow-up examination every 3 months for the first 2 years, then every 6 months for the next 3 years, and then every year thereafter. Follow-up examinations consisted of history taking, barium esophagography, physical examination, chest radiography, cervical ultrasonography, abdominal ultrasonography, and neck-abdomen computed tomography (CT) scans. If necessary, patients underwent positron emission tomography-CT and/or endoscopy. The study’s endpoint was cancer-specific survival (CSS), defined as the number of days from surgery to death caused by ESCC or the most recent follow-up visit. Data on CSS were collected from the follow-up records and verified by a special follow-up group. As of August 30, 2017, all patients in this study developed recurrence of LNs, previous surgical sites, and/or other organs. The median follow-up duration was 78.5 months.

### SCC-Ag and albumin levels and other data

The serum SCC-Ag and albumin levels were routinely measured at the SYSUCC clinical laboratory 1 week preoperatively. The baseline data included sex, age, smoking history, alcohol consumption, primary tumor location, and number of LNs.

### Statistical methods

All statistical analyses were performed using the SPSS software version 25.0 (IBM, SPSS, Inc., Chicago, IL, USA). All *P*-values were two sided, and a *P-*value < 0.05 was considered statistically significant. The associations between SCC-Ag level, albumin level, and clinicopathological characteristics were assessed using the χ^2^ test, Student’s t-test, and Fisher’s exact test. In addition, we used linear regression and Pearson’s correlation analysis to further explore the association between SCC-Ag and albumin levels. Standard deviation was used to evaluate the stability of continuous variables. Survival analyses were performed using life tables, Kaplan-Meier methods, and log-rank test. Cox regression and multivariate analyses were performed to identify whether SCC-Ag and albumin levels are independent prognostic factors in ESCC. In the previous studies, the X-Tile software (http://www.tissuearray.org/rimmlab v3.6.1) was used to determine the optimal cutoff value of some indicators [19, 20]. We estimated the C-statistics for survival data established by X-Tile software, which was similar to time-dependent Receiver Operating Characteristic curve analysis [21]. Therefore, we used the X-Tile software to reach the optimal cutoff levels of SCC-Ag and albumin. The preoperative serum SCC-Ag and albumin levels were considered high when they were > 1.0 μg/L and 39.8 g/L, respectively. Given that the SCC-Ag and albumin levels were continuous variables, patients were classified into two groups according to their concentrations (SCC-Ag, low level (0–1.0 μg/L, *n* = 249), High level (> 1.0 μg/L, *n* = 59); albumin, low level (0–39.8 g/L, *n* = 49), high level (> 39.8 g/L, *n* = 259)].

## Results

### Basic clinical characteristics of patients

Patients’ characteristics are summarized in Table 1. The study population consisted of 230 men (74.7%) and 78 women (25.3%) with a median age of 58.0 years (range, 33.0**–**75.0 years). The preoperative serum SCC-Ag level was low (≤ 1.0 μg/L) in 80.8% (*n* = 249) and high (> 1.0 μg/L) in 19.2% (*n* = 59) of the study cohort. There were 49 (15.9%) patients with low albumin level. Anastomotic leakage occurred in 28 patients during the perioperative period. The 1-, 3-, and 5-year CSS of all patients were 82.0, 69.0, and 65.0%, respectively. The median survival time of this cohort was 77.5 months.

**Table 1:** The associations between SCC-Ag, albumin level and the clinicopathological characteristics

Variables	All patients (*N* = 308)	SCC-Ag level			Albumin level		
		Low level (N = 249)	High level (N = 59)	*P* value	Low level (N = 49)	High level (*N* = 259)	P value
	No. of patients (%)/ mean ± std						
**Gender**				0.189^*^			0.883^*^
Male	230 (74.7%)	182 (73.1%)	48 (81.4%)		37 (75.5%)	193 (74.5%)	
Female	78 (25.3%)	67 (26.9%)	11 (18.6%)		12 (24.5%)	66 (25.5%)	
**Age** (year)	58.05 ± 8.34	57.41 ± 8.40	60.73 ± 7.59	0.006^**^	62.51 ± 8.40	57.20 ± 8.08	< 0.001^**^
**Smoking history**				0.608^*^			0.476^*^
No	108 (35.1%)	89 (35.7%)	19 (32.2%)		15 (30.6%)	93 (35.9%)	
Yes	200 (64.9%)	160 (64.3%)	40 (67.8%)		34 (69.4%)	166 (64.1%)	
**Drinking history**				0.857^*^			0.871^*^
No	217 (70.5%)	176 (70.7%)	41 (69.5%)		35 (71.4%)	182 (70.3%)	
Yes	91 (29.5%)	73 (29.1%)	18 (30.5%)		14 (28.6%)	77 (29.7%)	
**Tumor location**				0.344^***^			0.651^c^
Upper	24 (7.8%)	21 (8.4%)	3 (5.1%)		2 (4.1%)	22 (8.5%)	
Middle	102 (33.1%)	78 (31.3%)	24 (40.7%)		17 (34.7%)	85 (32.8%)	
Lower	182 (59.1%)	150 (60.3%)	32 (55.2%)		30 (61.2%)	152 (58.7%)	
**T stage**				0.017^***^			0.954^*^
T1	52 (16.9%)	49 (19.7%)	3 (5.1%)		9 (18.4%)	43 (16.6%)	
T2	84 (27.3%)	66 (26.5%)	18 (30.5%)		13 (26.5%)	71 (27.4%)	
T3	172 (55.8%)	134 (53.8%)	38 (64.4%)		27 (55.1%)	145 (56.0%)	
**Differentiation**				0.584^***^			0.494^***^
Grade I	77 (25.1%)	59 (23.7%)	18 (30.5%)		13 (26.5%)	64 (24.8%)	
Grade II	163 (53.1%)	132 (53.0%)	31 (52.5%)		29 (59.2%)	134 (51.9%)	
Grade III	66 (21.5%)	56 (22.5%)	10 (17.0%)		7 (14.3%)	59 (22.9%)	
Grade IV	1 (0.3%)	1 (0.8%)	0 (0%)		0 (0%)	1 (0.4%)	
**LNs**	20.0 ± 14.1	19.5 ± 13.6	26.8 ± 16.1	0.250^**^	17.2 ± 10.9	20.4 ± 14.6	0.146^**^
**Tumor length** (cm)	3.4 ± 1.7	3.3 ± 1.6	4.0 ± 1.6	0.002^**^	3.7 ± 2.0	3.4 ± 1.6	0.263^**^
**SCC-Ag** (μg/L)	0.80 ± 1.28	–	–	**–**	1.26 ± 2.23	0.72 ± 0.99	0.007^**^
**Albumin** (g/L)	43.2 ± 3.4	43.4 ± 3.1	42.3 ± 4.2	0.020^**^	–	–	–
**Anastomotic leakage**				0.855^*^			0.591^***^
No	280 (90.9%)	226 (90.8%)	54 (91.5%)		46 (93.9%)	234 (90.3%)	
Yes	28 (9.1%)	23 (9.2%)	5 (8.5%)		3 (6.1%)	25 (9.7%)	
**Chylothorax**				0.095^***^			0.406^***^
No	305 (99.0%)	248 (99.6%)	57 (96.6%)		48 (98.0%)	257 (99.2%)	
Yes	3 (1.0%)	1 (0.4%)	2 (3.4%)		1 (2.0%)	2 (0.8%)	
**Respiratory complications**				0.254^***^			0.699^***^
No	296 (96.1%)	241 (96.8%)	55 (93.2%)		48 (98.0%)	248 (95.8%)	
Yes	12 (3.9%)	8 (3.2%)	4 (6.8%)		1 (2.0%)	11 (4.2%)	
**Cardiovascular complications**				1.000^***^			0.245^***^
No	301 (98.0%)	243 (98.0%)	58 (98.3%)		47 (95.9%)	254 (98.4%)	
Yes	6 (2.0%)	5 (2.0%)	1 (1.7%)		2 (4.1%)	4 (1.6%)	

*: χ^2^ test; **: student’s t test; ***: Fisher’s exact test; LNs: the number of lymph nodes removed in the surgery

### Correlations between SCC-Ag and albumin levels and clinicopathological characteristics

**Fig. 2:**
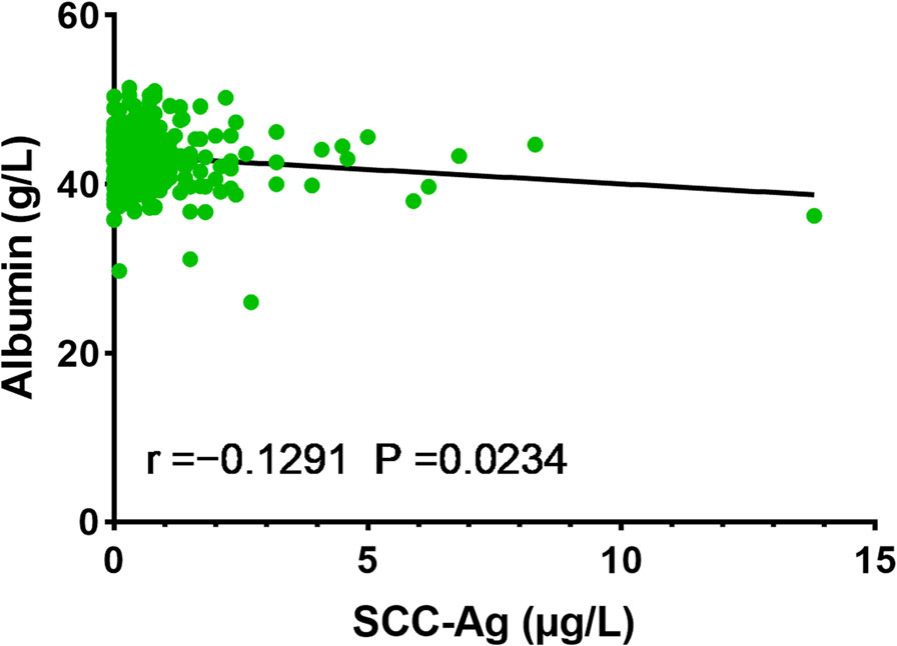
Correlation between SCC-Ag and albumin levels

Table 1 shows the relationships between the SCC-Ag and albumin levels and various clinical and pathological features. We found that pathological T stage, age at diagnosis, length of tumor, and albumin level were likely to be associated with SCC-Ag level (all *P* < 0.05). In addition, SCC-Ag level and age at diagnosis were related to preoperative albumin level (all *P* < 0.05). We also found that the SCC-Ag level had a significant negative correlation with albumin level (*P* = 0.0234, Fig. 2) by Pearson’s correlation analysis. However, the correlation was extremely weak (r = − 0.1291).

### Univariate and multivariate analyses

Univariate and multivariate Cox regression analyses (Table 2) indicated that the five independent prognostic factors in patients with ESCC were alcohol consumption, pT staging, SCC-Ag level (adjusted hazard ratio [HR], 1.200; 95% confidence interval [CI], 1.083–1.330; *P* = 0.001), LNs, and albumin level (adjusted HR, 0.945; 95% CI, 0.896–0.997; *P* = 0.038).

**Table 2:** Univariate and multivariate Cox regression analysis for cancer specific survival in patients ESCC with stage T1-3N0M0 (Cox regression’s method is Forward: LR)

Characteristics	Univariate analysis			Multivariate analysis		
	HR	95% CI	*P* value	HR	95% CI	*P* value
**Gender** (male vs. female)	0.722	0.456–1.141	0.163			
**Age** (continuous)	1.020	0.997–1.043	0.082			
**Smoking history** (no vs. yes)	1.608	1.059–2.443	**0.036**	–	–	0.283
**Drinking history (**no vs. yes)	1.681	1.150–2.459	**0.007**	1.878	1.275–2.765	**0.001**
**Primary tumor location**						
Upper (reference)	1	–	–			
Middle	0.728	0.334–1.585	0.424			
Lower	1.023	0.688–1.522	0.910			
**pT stage** (T1 vs. T2 vs. T3)	1.445	1.102–1.894	**0.008**	1.551	1.167–2.061	**0.002**
**SCC-Ag** (continuous)	1.227	1.105–1.361	**< 0.001**	1.200	1.083–1.330	**0.001**
**Albumin** (continuous)	0.939	0.889–0.991	**0.022**	0.945	0.896–0.997	**0.038**
**LNs** (continuous)	0.984	0.969–0.999	**0.040**	0.982	0.967–0.997	**0.020**
**Tumor length** (continuous)	1.070	0.960–1.194	0.222			
**Anastomotic leakage** (no vs. yes)	1.078	0.579–2.009	0.812			
**Chylothorax** (no vs. yes)	1.066	0.149–7.640	0.949			
**Respiratory complications** (no vs. yes)	0.880	0.324–2.389	0.803			
**Cardiovascular complications** (no vs. yes)	1.118	0.276–4.529	0.876			

The factors in the univariate analyses with *P* value less than 0.05 would be took in account into multivariate analyses. *LNs* the number of lymph nodes removed in surgery, *HR* hazard ratio, *CI* confident interval

### Survival outcomes of patients

**Fig. 3:**
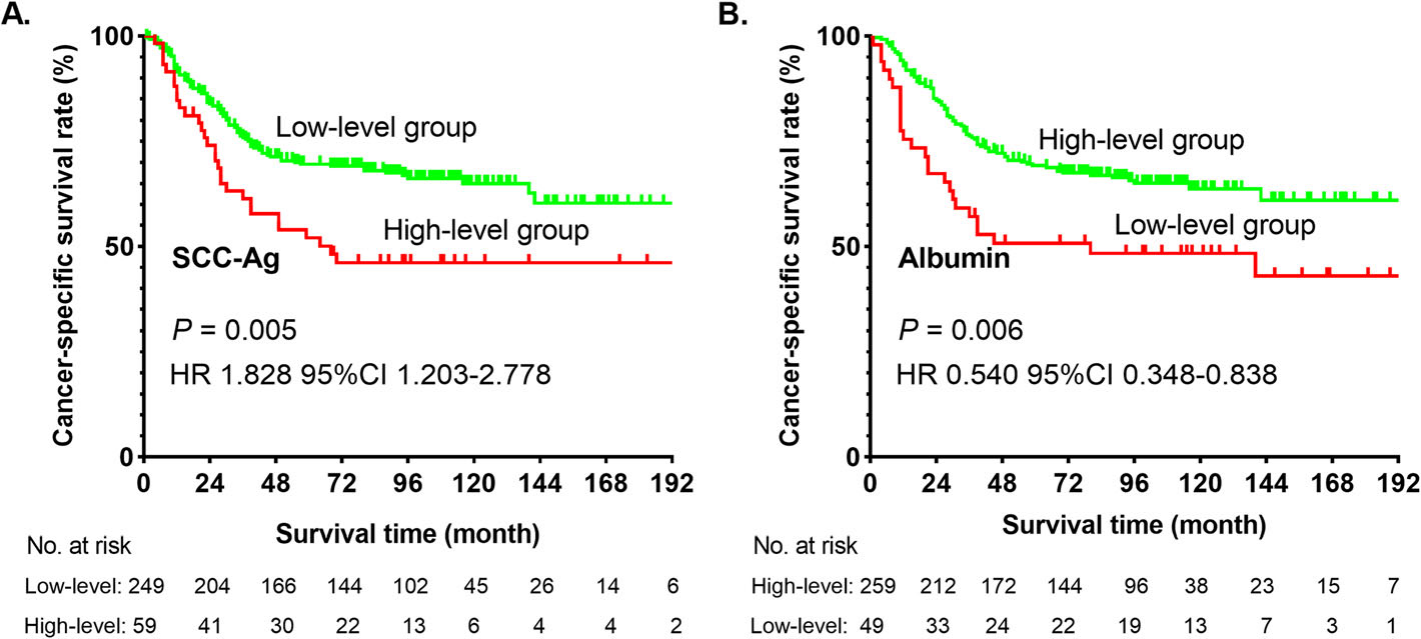
Cancer-specific survival curve for the whole cohort of patients with ESCC with stage T1-3N0M0 according to the preoperative serum SCC-Ag (**a**) and albumin (**b**) levels

Kaplan-Meier method demonstrated that a high serum SCC-Ag level (*P* = 0.005) or low serum albumin level (*P* = 0.006) were associated with low CSS rate in patients with ESCC (Fig. 3). Moreover, life tables revealed a difference in 1-, 3-, and 5-year CSS rates between the low and high SCC-Ag level groups (1-year CSS, 84.0% vs 74.0%; 3-year CSS, 71.0% vs. 58.0%; 5-year CSS, 69.0% vs. 46.0%). The 1-, 3-, and 5-year CSS rates in patients with low albumin level were lower than those with high albumin level (1-year CSS, 67.0% vs 85.0%; 3-year CSS, 51.0% vs. 72.0%; 5-year CSS, 51.0% vs. 67.0%).

**Fig. 4:**
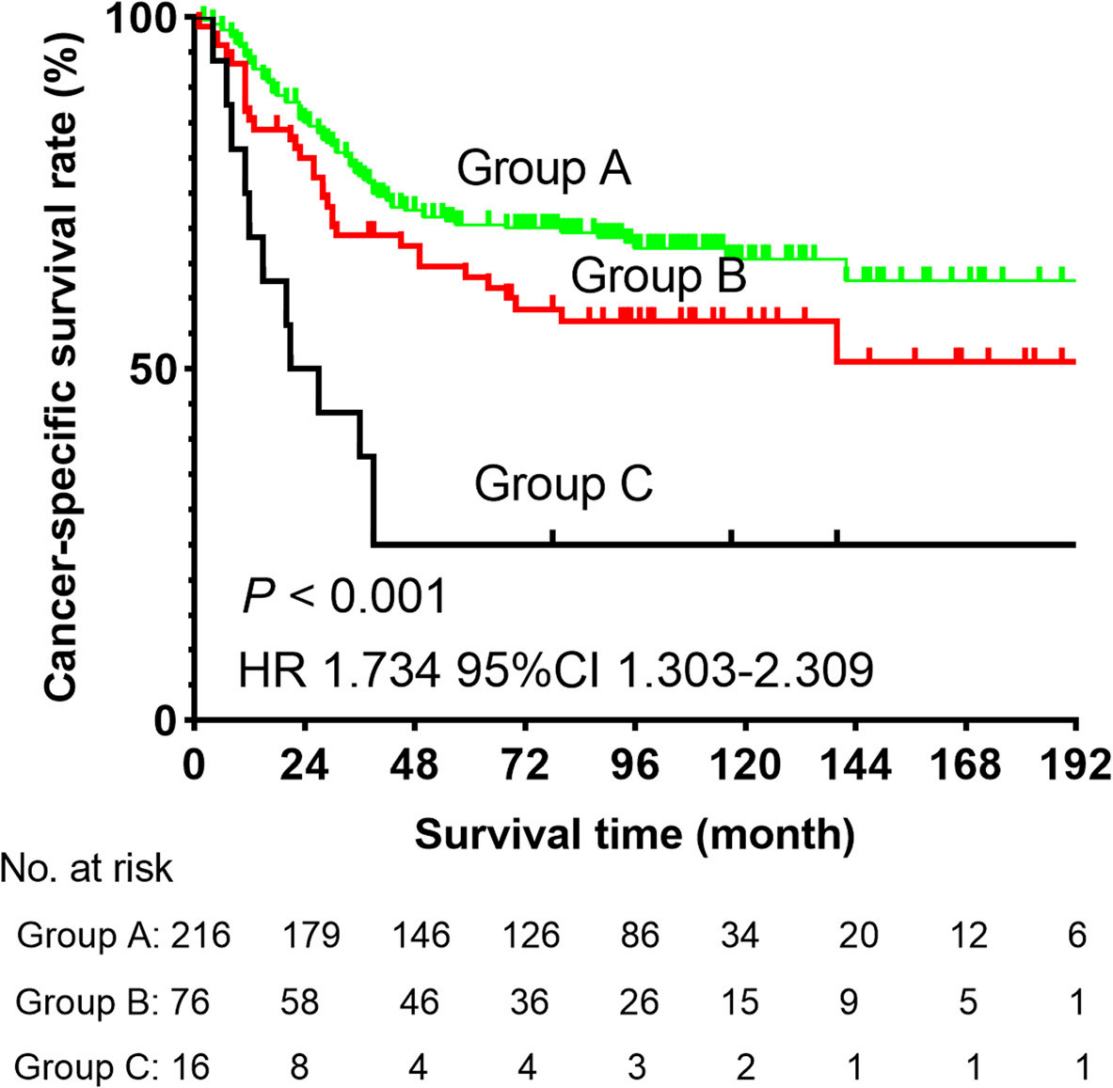
Cancer-specific survival curve for the whole cohort of patients with ESCC according to the groups (Group A included low SCC-Ag and high albumin levels; Group B included patients with high SCC-Ag or low albumin levels; Group C included patients with low albumin and high SCC-Ag levels)

We combined the SCC-Ag and albumin levels and classified patients into three groups (Group A included patients with low SCC-Ag and high albumin levels; Group B included patients with high SCC-Ag or low albumin levels; Group C included patients with low albumin and high SCC-Ag levels). We found that Group C had the worst survival than other groups (all *P* < 0.005, Fig. 4). The 5-year CSS rate in Groups A, B, and C were 70.0, 58.0 and 25.0%, respectively.

## Discussion

Many factors including smoking history and alcohol consumption were reported to predict ESCC prognosis [22–24]. In this retrospective study, we explored the prognostic role of preoperative SCC-Ag and albumin levels in a large sample of 308 patients with ESCC with stage T1-3N0M0. We analyzed the data of these patients. Eventually, we found that SCC-Ag and albumin levels could be prognostic predictive indicators. In addition, albumin level was likely to be a protective prognostic factor, but SCC-Ag level might be a risk factor (Table 2). This study was the first to combine SCC-Ag and albumin levels to further analyze prognosis of patients with ESCC with stage T1-3N0M0. The results showed that patients with low albumin and high SCC-Ag levels had the worst 5-year CSS than patients in the two other groups (Fig. 4). Given that the information of preoperative SCC-Ag and albumin levels can be conveniently obtained from medical records, our findings had a certain application value. Based on the survival outcomes observed in this study, patients with a high preoperative SCC-Ag level and low albumin level can be advised to undergo more active treatment, such as adjuvant chemotherapy and radiotherapy, to achieve better CSS.

More than 90% of esophageal cancers in China are of squamous carcinoma type, and serum SCC-Ag level had been highly recommended and could be a critical biomarker for prognosis. In a previous study in Japan, the cutoff SCC-Ag level was 1.5 μg/L, and the positive rate of SCC-Ag in resected ESCC was 25% [15]. In our study, the cutoff level determined using X-Tile program was 1.0 μg/L, and the positive rate was found to be lower, at 19.2%. This difference could be attributed to disparity of TNM stage and our low SCC-Ag cutoff level (1.0 μg/L). The varying surgical indications among hospitals may have also caused sampling bias. In clinical practice, the optimal cutoff SCC-Ag level to determine high-risk patients with ESCC remains controversial. The widely used level of 1.5 μg/L was based on an overall population; however, it is not likely to reflect the risk of prognosis in patients with ESCC. Our study explored a new cutoff SCC-Ag level (1.0 μg/L), and the two groups that were grouped based on this level showed a strong difference in survival (*P* = 0.005). In this study, we observed that the SCC-Ag level adjusted for tumor staging was an independent prognostic factor for patients with ESCC, similar to the results of Shimada et al. [15, 25]. The most likely explanation was that the SCC-Ag level is mainly determined by tumor volume and internal potential for malignancy, such as expression of gene Wnt-11 [11, 26], which cannot be well described by the TNM staging system. For example, as for T staging of ESCC, we only consider the depth of tumor invasion rather than tumor size or volume. Similarly, in our study, SCC-Ag level was considered as an independent prognostic factor adjusted for pT stage (Table 2).

In addition, in the field of ESCC, a previous study suggested that the Glasgow Prognostic Score (GPS) could be a prognostic factor for disease-free survival [27]. Of note, the GPS system included albumin and C-reactive protein levels, and the observed endpoint of Matsuda’s study was different from ours [27]. In another study, Han et al. collected the information of 101 patients with esophageal adenocarcinoma and found that patients with albumin level ≥ 40.0 g/L had better overall survival by analyzing their data (*P* < 0.05) [28]. Similarly, we illustrated that patients with albumin level > 39.8 g/L had more survival advantage than patients with albumin level ≤ 39.8 g/L. However, there were some differences between our study and Han’s study, such as observed endpoint and pathological types of esophageal cancer. Therefore, it is significant to explore the effect of preoperative albumin level on CSS of patients with ESCC with stage T1-3N0M0.

As a retrospective study, this had certain limitations. First, the patients were unevenly distributed in each T stage, and this may have affected our results. To the best of our knowledge, ESCC patients with metastasis of LNs have a poor survival outcome. In fact, the status of LNs may reflect the malignancy of ESCC and affect the serum SCC-Ag level. However, patients with LN-positive ESCC were not included in this study. Accordingly, a large sample size is needed for further analysis. In addition, we are likely to recruit patients with metastasis of LNs to further explore the association between the status of LNs and SCC-Ag level. Second, our follow-up data lacked the regular surveillance of postoperative SCC-Ag and albumin levels, restricting evaluation of the role of postoperative SCC and albumin levels on long-term follow-up monitoring of patients with ESCC. Postoperative SCC-Ag level, albumin level, and detailed follow-up, including tumor recurrence and metastasis status, are also vital in analyzing the prognostic roles in patients with ESCC with stage T1-3N0M0. Third, the cutoff SCC-Ag level (1.0 μg/L) and albumin level (39.8 g/L) determined by X-Tile software are likely to be affected by “human factors.” We suggested that SCC-Ag and albumin levels might be independent prognostic factors by analyzing continuous values (Table 2) and then stratifying the patients using the abovementioned cutoff levels. However, there are still some differences in cutoff levels between our study and other studies. Therefore, appropriate statistical methods and adequate sample size are needed to further determine the optimal cutoff levels.

## Conclusions

The preoperative SCC-Ag and albumin levels, apart from the TNM staging system, were independent prognostic factors for ESCC in stage T1-3N0M0. In addition, patients with low albumin and high SCC-Ag levels had worst 5-year CSS than other patients. However, further study is needed to explore the impact of SCC-Ag and albumin levels on the prognosis of patients with ESCC with stage T1-3N0M0.

## Data Availability

Please contact author for data requests. The data of this study have loaded in the Research Data Deposit (No. RDDA2020001402, www.researchdata.org.cn).

## Abbreviations

SCC-Ag: squamous cell carcinoma antigen
CT: computed tomography
CSS: cancer-specific survival
ESCC: esophageal squamous cell cancer
HR: Hazard Ratio
CI: Confidential Interval
SYSUCC: Sun Yat-sen University Cancer Center
AJCC: American Joint Committee on Cancer
LNs: lymph nodes
std.: standard deviation
